# The Truth Shall Make Us Free

**Published:** 1895-06

**Authors:** J. H. Allen

**Affiliations:** Logansport, Ind.


### THE TRUTH SHALL MAKE US FREE.

[SECOND ARTICLE.]

J. H. ALLEN, M. D., LOGANSPORT, IND.

What is truth? says the prejudiced mind, but will not stay for an answer. He would rather drift along in his empiricism than to investigate the workings of some immutable law, and be a slave (as he thinks) to some fixed principle of the universe; or, in other words, he will not let God work through him. The world is and ever has been fighting for the truth, and as far as our mental grasp goes, it is an honest war; but the greater majority of our medical friends seem to believe in the philosophy of the Greeks,* and have come to the conclusion, as far as therapeutics is concerned, that we have no criterion by which to form a judgment.

The fact is, if we could but eliminate our vain opinions and vain imaginations, and, as one great writer puts it, "clothed in our right mind and wearing the garb of truth," we are then in a condition to sail into the undiscovered ocean of thought and dip farther into the future than mortal eyes have yet seen. Bacon says: "It is not the lie that passes through the mind, but the one that sinketh into it and settleth there, that hurts."

Truth, as I have said in a previous article, brings light; but it does more than that—it brings good; and it was Socrates who said: "Good is that which brings us the most lasting benefits." So in our application of Homœopathy; it is something from which we get a more lasting benefit, and in its study we find that its developments are good and its laws enable us to

---

* New Academy.

overcome those unseen forces, those morbid influences, known as disease, by drawing upon the thousand and one of the therapeutic forces which Nature has so bountifully provided to overcome them, through the powers of similia. The practitioner of medicine who has not conceived this philosophical truth is like a man sailing upon an ice floe—subject to the shifting winds and changing currents; if he puts his vain imaginations first, and, not like a Newton or a Hahnemann, who, in their earnest search after truth, lost sight of themselves, and, dwelling in the spirit of truth, were governed by supreme law. This is the position every follower of Hahnemann must assume in order to understand the truth and have a clear conception of the law of similia.

We must take ourselves out of the paths of divine circuit or let in the analytical light of the law of Homœopathy, that we may know what is the wisdom of God by a knowledge of His laws. He always works through them, so we may know where to find Him. Now, knowing this truth we become an Archimedes, drawing from the central forces of power by our humble obedience to its demands; but being without law we are simply powerless, a mere nothing, a vessel beached upon the sands, void of power. In fact, our ability in any sphere in life is in proportion as we comply with, or, in other words, as we draw upon, law. To be governed by principle is to be governed by truth; to be governed by truth is to be governed by law; and to be governed by law is to be governed by Omnipotence. "A higher law than man's reason regulates events," and it is only in our easy, simple, and spontaneous actions that we are strong. To have a law that we believe in lifts from us a great load, and in place of leaning upon ourselves we lean upon law, and obedience to it leads up to higher and better things. We at once become students of nature, studying the phenomena of natural laws, and scientists in the laboratories of the force world, comparing the laws of biogensis with the laws of similia, so that every new development makes us stronger in our faith. Law is spiritually conceived, and it is not every one who runs can read. It speaks to us in a voice so very low, yet loud enough to be heard by the humble listener, and in the language that is foreign to the ear of the unbeliever, and in a cipher that can only be found in similia, simplex, minimum.

The application of law to disease or to the disturbed life forces calls into effect the forces governed by that law; so you see we may use any force that comes under the government of that system. And when we speak of system we are brought into the presence of a colossus, and we gaze in mental awe at the greatness of its magnitude. At once we see that it is infinite, and being infinite it is eternal. Year by year, as earnest students of its phenomena, we incorporate under our jurisdiction more and more of its power, until in time we shall dismiss from our therapeutics all expedients and depend entirely on the power and richness of similia. It is a part of God's plan to let the human mind find out the truth for itself; and it is a pleasure, a privilege, and a duty for each one to investigate for himself.

Truth, we are told, is an exact correspondence of subjective and objective relations. In common life we call truth an agreement of the object and our conception of the object. Thus we presuppose an object to which our conception must conform. Philosophically speaking, truth is an agreement of the subject-matter of thought with itself. It is the highest quality of science, of art, of philosophy, and of law. It never deflects or bends, but is as immutable as law itself. The study of its phenomena lies to a great degree within our reach, and we must at all hazards push out our inquiries. "Search," is the command, and the reward is, we shall find. Though it is well to lean upon the promise, it is wisdom to obey the command.

We look upon men who make new discoveries in any department of science as being in some way inspired or endowed with some supernatural power; as, for instance, the astronomer is inspired by his work, or the poet by his muse; when the truth of the matter is, the true scientist recognizes that he is standing in the omnipresence of law, and it has taken full possession of him and is guiding and directing him, yea, leading him into those mysterious by-ways were thought itself scarce dare to enter, only to find the golden gates fly open at his approach and reveal their hidden treasures in the richness of their pro-

18

fusion: treasures that had been locked under the time-lock of ages, and, like Hahnemann, given to them as a rich legacy, and to be passed to their kindred yet unborn.

We must deal with life on principles of the clearest character; principles not human, therefore not limited. These principles are not approachable by ordinary thought. Truly we are in ignorance of what lies behind life. Behind that thin yet seemingly impenetrable veil is hidden all science, all art, all philosophy, all the forces, all the powers of which we as mortals are endowed; and we cannot open with a golden key these golden doors and peer ruthlessly within. It is only by a knowledge of the law governing these things that we are led up from hyperbole unto hyperbole; and as soon as we are in possession of one truth it opens the highway to another, and so on without end, as it was in the case of Hahnemann. The law of similia was born from a single truth, conceived in the actions of China or Cinchona and its similarity to malaria poison. Thus it ever must be, one truth can only be born of another truth. Then we began with truth which brought us light, that discovered for us law, which gave us true knowledge, that developed good, and that brought us a more lasting benefit. The value of any truth depends upon the amount of good we can get out of it. If it does not exalt men and make them better either physically, mentally, morally, socially, or intellectually drop it; do not bother with it; do not worry your brains over it, for it is of no consequence; but if it is a great truth, like Homœopathy, founded on law, a divine gift of which nothing can come but good; if this be true let us centre our minds upon it; let us focus our intellectual lenses upon it and pry deep into the mysteries and learn more of it. Giliad's balm can be found in Aconite, in Apis, in Aloes, in Arsenic and a thousand and one things; then let us not be mosaics of other men but let us be a new conception, a new thought, for other men to construct the ground-plan for grander and better things. When we say men are geniuses or scientists or philosophers we do not mean that they are giants of intellect or that their minds have increased to that volume by which they may overpower other men, but that they have pushed out into the unknown, and the veil was rent and the gates that were closed against you and me opened to the gentle touch of the earnest searcher after truth. So you perceive that truth rends the veil between the known and the unknown. Oh! what a power we have in the application of law in subduing elements or in combining them! almost a creative power. Through law we have control over the material and harness the forces of nature, or bind them in statics or let them loose in energy. By diving down into the soul of things we are enabled to awaken a spirit of research in others, so that they become interested with us and the things that were a mystery to us before we had any knowledge of the law now become intelligible, and we become viewers of nature through the telescope of law, which gives us a genetic view of things, untrammeled by prejudice or any aberration of a mind that is not governed by a true principle.

Then let us push forward and onward into the very avenues of truth. Tantalus is shining and rippling before us; why not reach out and drink of it; why not sail on to the head-waters of truth, and not as the slow ebbing of a receding tide, drop unconsciously back into oblivion?

[TO BE CONTINUED.]

